# Multivariate Porous Aromatic Frameworks with High Porosity and Hierarchical Structures for Enzyme Immobilization

**DOI:** 10.1021/acscentsci.3c00078

**Published:** 2023-02-16

**Authors:** Zhaofu Zhang, Yajing Zheng, Zilong Dou, Mengnan Gu, Mengxiao Sun, Jian Song, Nan Gao, Fengchao Cui, Yuyang Tian, Guangshan Zhu

**Affiliations:** Key Laboratory of Polyoxometalate and Reticular Material Chemistry of Ministry of Education, Faculty of Chemistry, Northeast Normal University, Changchun, 130024, China

## Abstract

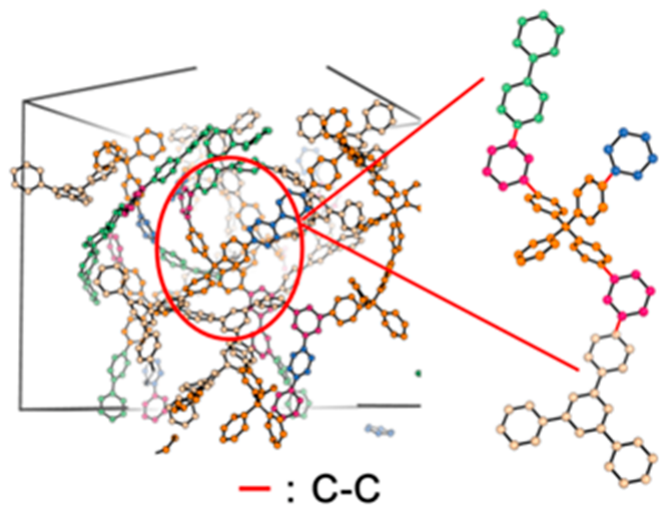

As materials with permanently porous structures and readily modifying availability, porous aromatic frameworks (PAFs) are considered as promising porous materials with versatile functionality. Currently the designable synthesis of PAFs with the desired surface area and pore size is still a challenge, and instead kinetically irreversible coupling reactions for PAFs synthesis has resulted in the unpredictable connection of building units. Herein, a series of PAFs with highly porous and hierarchical structures were successfully synthesized through a multivariate inspired strategy, where multiple building units with various topologies and sizes were selected for PAFs synthesis. All the PAFs synthesized through this strategy possessed hierarchical structures and high specific surface areas at the same time. Encouraged by their high surface area and hierarchical structures, we loaded lipase onto one of the multivariate PAFs. The enzyme loading content of the obtained lipase@PAF-147 was as high as 1456 mg g^–1^, which surpassed any other currently reported enzyme loading materials. The lipase@PAF-147 also exhibited favorable catalytic activity and stability to a model reaction of *p*-nitrophenyl caprylate (*p*-NPC) hydrolysis. This multivariate strategy inspired synthetic method broadens the selection of building units for PAFs design and opens a new avenue for the design of functional porous materials.

## Abstract 2

Here, we synthesized a new series of porous aromatic frameworks (PAFs) by multiple building units based on a multivariate strategy. These PAFs had a high surface area, hierarchical structure, and highly porosity, and could immobilize enzymes with high content.

## 1 Introduction

Porous materials are widely used in vast research fields such as adsorption, separation, catalysis, drug delivery, etc.^1−3^ For specific usage, the designed synthesis of desired porous structures and pore chemistry is always pursued. For example, catalytic process catalysts with a high surface area provide abundant accessible interfaces for adsorption, and their opening pores at molecular scale selectively accommodate specific molecules depending on their shapes and sizes. In the past few decades, metal–organic frameworks (MOFs) and covalent organic frameworks (COFs) have been developed.^4,5^ The reticular synthesis by building unit design and oriented coordination/covalent bond connection renders their high porosity with desired framework structures, but the relatively weak coordinating bonds or reversible covalent bonds still limit their stability for further utilization. In contrast, porous organic materials constructed by kinetically irreversible coupling reactions possess high stability and structural maneuverability. Many kinds of porous organic materials have been reported and studied,^6^ and porous aromatic frameworks (PAFs) are one representative of them.^7^ In PAFs structures, the aromatic ring based building units are linked by carbon–carbon bonds, so PAFs are resistant to harsh chemical treatments.^8^ The excellent chemical stability and the aromatic ring confer PAFs good chemical amenability, and thus PAFs show significant potential in applications including gas separation, catalysis, adsorption, etc.^7,9−13^

Although being able to form stable carbon–carbon linkage, kinetically irreversible coupling reactions for PAFs synthesis also bring significant difficulties in controlling their growing process. As a result, the synthesis of highly porous PAFs remains a challenge and is often achieved by chance. Although a series of PAFs have been reported so far, only a few materials showed a high specific surface area over 2000 m^2^ g^–1^.^8,14,15^ Meanwhile, building units for the construction of high surface area PAFs are limited. According to statistics, PAFs with a high specific surface area were almost synthesized exclusively from tetrahedral building units or their derivatives,^15^ which was possibly due to the insufficient alignment of rigid tetrahedral building units in space. However, building units with other topological connecting shapes such as linear or triangular would lead to decreased yield and a substantial decrease of the specific surface area.^16,17^ This was explained by the framework interpenetration in several researchstudies,^18,19^ but other reasons may also be attributed to this fact, such as the uneven dispersion or agglomeration of building units, and also the undesired connection of linear building units to the formation of soluble and polymer-like molecules, other than extended frameworks. Therefore, the limitation in selecting appropriate building units for highly porous PAFs resulted in a limited structural complexity.

Hierarchical porous materials are especially desired in the field of biocatalysis, because the hierarchical skeleton with mesoporous provides enough space to accommodate enzyme molecules. At the same time, the highly porous framework promotes the diffusion of the catalytic substrates.^20^ Until now, many MOFs and COFs with a high surface area and mesoporous structures have been used for enzyme loading and catalysis.^21−24^ However, for the PAFs, using large building units to construct mesoporous structure not only faced the problem of framework interpenetration that decreases the pore size, but also the porosity was significantly degraded due to the undesired connection of building units. Moreover, a trade-off between the surface area and pore diversity of PAFs was usually observed. PAF-1 with a diamond structure has a surface area of 5600 m^2^ g^–1^, which was the highest among all the reported PAFs,^7^ but attempts to expand the pore size with an isoreticular structure led to a dramatic decrease of its surface area.^25^ Due to the uncontrollable synthetic process, the synthesis of PAFs with a high specific surface area and hierarchical structures has not been widely reported so far.

Herein, in order to expand the applicability of building units with various shapes and sizes, a synthetic strategy to multivariate PAFs (MTV-PAFs) was proposed. Multivariate building units were used for the synthesis of MOFs and COFs to introduce structural complexity or functional heterogeneity.^26−28^ Similarly, multiple building units were used for the synthesis of MTV-PAFs. PAF-147, PAF-148, and PAF-149 were synthesized with five distinct building units under varied ratios. In the synthesis of MTV-PAFs, it is probably the entropy increment that drives the even distribution of multiple building units and leads to the increased connecting possibility of forming highly cross-linked structures.^29^ The results showed that all of the MTV-PAFs obtained in this work exhibited a high specific surface area above 2800 m^2^ g^–1^, and they all have hierarchical structures with a mesopore size at 4–5 nm. Encouraged by their structural features, we selected a lipase from *Aspergillus oryzae* for enzyme immobilization, and found the loading capacity for PAF-147 was as high as 1456 mg g^–1^, which surpasses the contents of any other mesoporous materials reported to date. Meanwhile, the immobilized enzyme was highly resistant to temperature and pH changes, and the catalytic activity was well recycled. These results have demonstrated that this multivariate inspired synthetic strategy is a feasible route to PAFs with high porosity and a specific porous structure.

## 2 Results and Discussion

**Figure 1:**
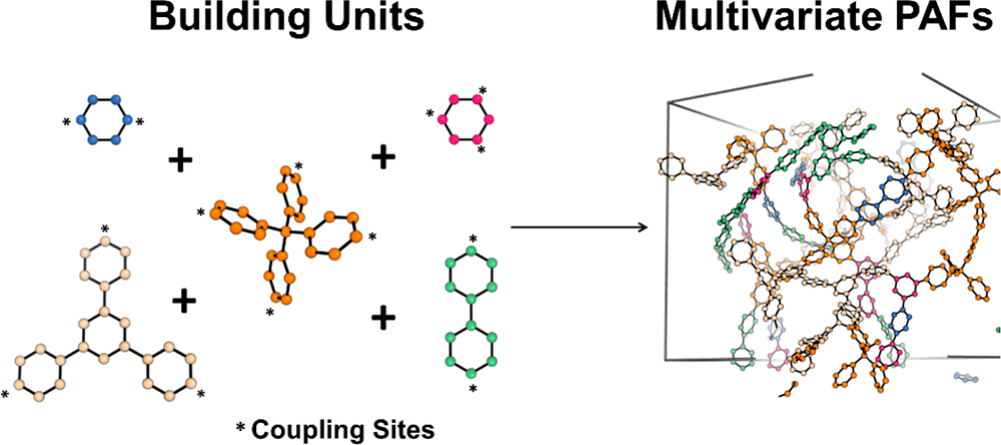
Synthesis schematic of the MTV-PAFs and a diagrammatic sketch. The asterisk represents the sites where the coupling reaction occurred.

The synthetic route to the MTV-PAFs was shown in Figure 1, and the synthetic details can be found in the Experimental Section. Five molecules with different shapes and sizes [1,4-dibromobenzene, 4,4-dibromobiphenyl, 1,3,5-tribromobenzene, 1,3,5-tribromophenylbenzen, and tetra(4-bromophenyl) methane] were selected as building units, and varied ratios of building units gave PAF-147, PAF-148, and PAF-149, with their yields all above 85% (details shown in Table S1). The successful syntheses of these PAFs were demonstrated by FT-IR spectra. As shown in Figure S1a,b, the C–Br stretching vibration of five building units at the wavenumber of 512, 532, and 1078 cm^–1^ disappeared in the spectra of PAF-147, PAF-148, and PAF-149, indicating the coupling reactions among these building units successfully occurred to form carbon–carbon bonds in PAFs network as expected. The results of elemental analysis are shown in Table S2, with the C element ratios of PAF-147, 148, and 149 to be 93.14%, 90.12%, 91.12%, and the H element ratios 2.44%, 3.08%, 3.84%, respectively. The remaining fraction was assigned to the oxygen element from remaining guest molecules which was not detectable by the instrument. Thermogravimetric analysis is shown in Figure S2. The weight loss curves of PAF147, PAF-148, and PAF-149 over increased temperature revealed two stages under air flow (Figure S2a). In the temperature range of 0–450 °C, the weight loss was about 4–7% due to guest removal, and in the range of 450–600 °C a 91–95% weight loss occurred, which was mainly attributed to the decomposition of the carbonous framework. Almost no residual was found after calcination, indicating no inorganic catalyst left in the frameworks. Additionally, PAF147, PAF-148, and PAF-149 were examined by SEM, and they were all found to be spherical nanoparticles with particle sizes ranging from 100 to 300 nm (Figure S3).

**Figure 2:**
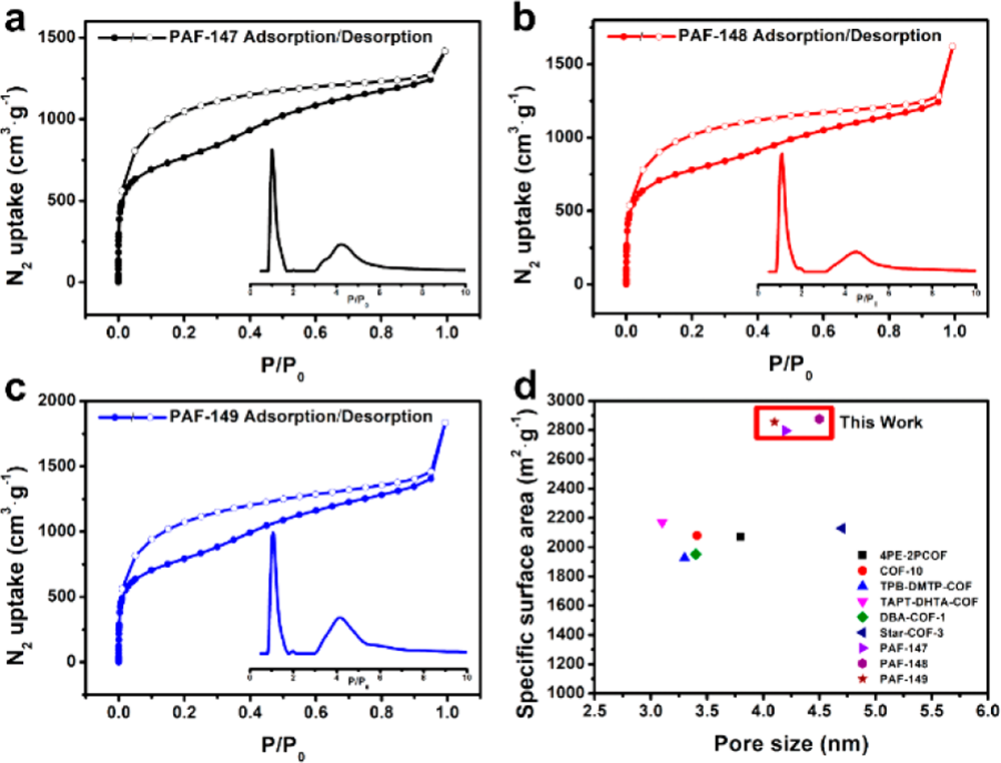
N_2_-sorption isotherm and pore-size distribution of **a**, PAF-147, **b**, PAF-148, **c**, PAF-149 and **d**, a plot diagram of a specific surface area versus pore size of mesoporous materials with 3–5 nm pores; data sources are listed in the Supporting Information Table S6.

The porous features of the MTV-PAFs were determined by nitrogen adsorption measurements at 77 K. All the PAFs were degassed at 80 °C for 12 h before testing. As shown in Figure 2a–c, the nitrogen sorption isotherms displayed sharp uptakes at low relative pressure, and hysteresis loops at higher relative pressure, which indicated that there were both microporous structures and mesoporous structure in PAF-147, PAF-148, and PAF-149. The BET surface areas of PAF-147, PAF-148, and PAF-149 are calculated to be 2797, 2877, and 2856 m^2^ g^–1^, respectively. Pore size distribution calculated by the nonlocal density functional theory (NLDFT) indicated these materials had narrowly distributed micropores and mesopores at 1.0 and 4.2 nm for PAF-147, 1.0 and 4.5 nm for PAF-148, 1.0 and 4.2 nm for PAF-149. The N_2_ adsorption also indicated the pore volumes for PAF-147, PAF-148, and PAF-149 are 1.9 cc g^–1^, 2.0 cc g^–1^, and 2.2 cc g^–1^, respectively (Table S4). These results indicated that PAFs with high specific surface area and hierarchical structure were successfully synthesized by the construction of multiple building units, and these building units with different topological shapes and sizes contributed to their porosity and pore size. In order to demonstrate that the multiple building units played an important role in the synthesis process, two materials (Compound 1, Compound 2) which were synthesized from only two building units at equal molar ratios were prepared and characterized. The synthetic processes were as same as that of MTV-PAFs, as detailed in the Supporting Information (Figures S1c, S2b, S4, and S5). First of all, it was found that the yield for Compound 1 was only 50%. In the aforementioned discussion, the relatively low yield compared to the MTV-PAFs was probably due to the formation of soluble polymer like molecules when linear building existed. It was also found that the BET surface areas of Compound 1 and Compound 2 were 1185, 2218 m^2^ g^–1^, respectively, and their pore size distribution centered at 1.41 nm for Compound 1, and 1.68 and 2.24 nm for Compound 2. Compared to the MTV-PAFs, these surface areas were lower, and the combination of only two building units formed microporous frameworks, indicated by their N_2_ adsorption isotherms and pore size distribution (Table S5). The difference suggested that the entropy effect controlled the highly disordered distribution of the multiple building units in the synthetic system.^29^ When multiple building units existed, they evenly coupled and connected with each other, which facilitated the formation of a structure with high porosity. Meanwhile, linear building units with a large size contributed to the formation of mesopores to give a hierarchical structure. As a result, the statistics suggested that PAF-147, PAF-148, and PAF-149 exhibited high specific surface areas among all the reported porous materials with 3–5 nm pores (Figure 2d and Table S6); more details could be found in the Supporting Information.

**Figure 3:**
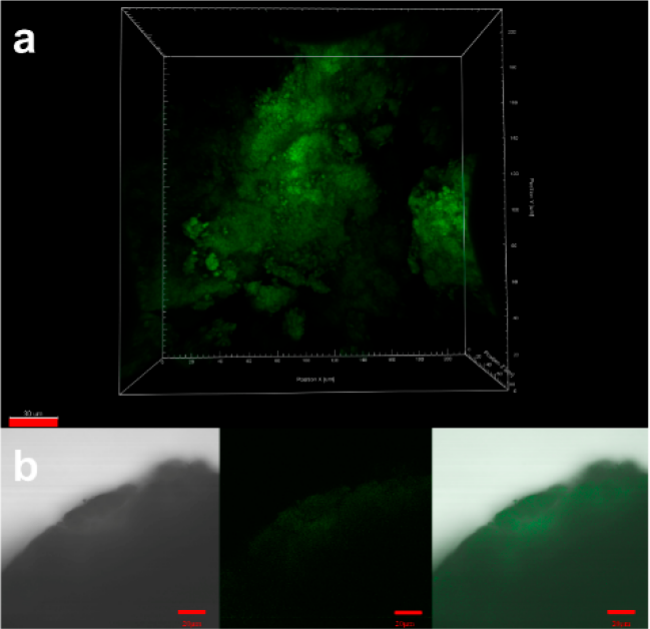
**a**, the 3D CLSM image of lipase@PAF-147, scale bar: 30 μm and **b**, the 2D CLSM images of lipase@PAF-147 scale bar: 20 μm (left: white light, middle: fluorescence, right: white light + fluorescence).

**Figure 4:**
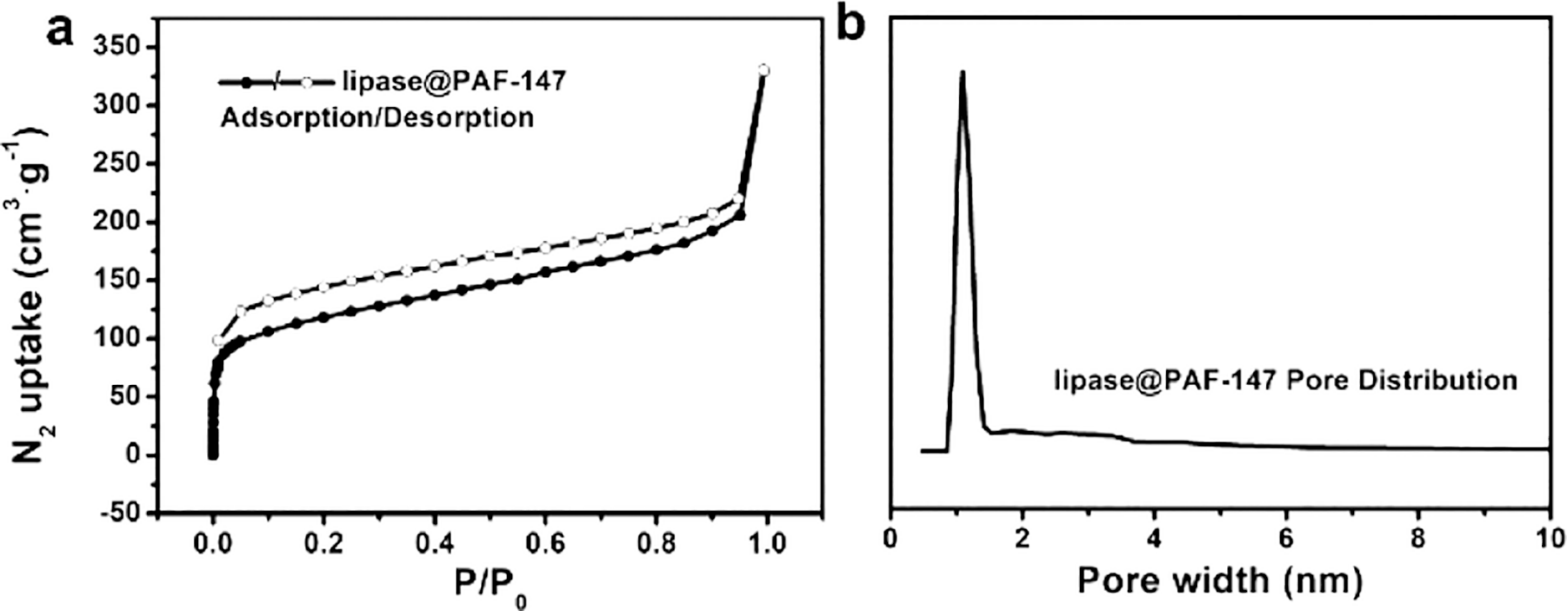
(a) The N_2_-sorption isotherm of lipase@PAF-147 and (b), the pore-size distribution of lipase@PAF-147.

Encouraged by the high surface area and hierarchical structure of PAF-147, -148, and -149, we consider these materials to be promising for enzyme loading. Lipase from *Aspergillus oryzae* (4.4*4.7*4.8 nm) was chosen as a model compound due to its suitable size and usage as an effectively catalyst for the hydrolysis of *p*-nitrophenyl caprylate (*p*-NPC) to 4-nitrophenol. Due to the similarity in the surface area and pore size of PAF-147, PAF-148, and PAF-149, only PAF-147 was selected as the carrier material for enzyme loading. The lipase@PAF-147 was characterized and its catalytic performance was investigated. First, the amide bond of lipase at the wavenumber of 1648 cm^–1^ appeared in the spectrum of lipase@PAF-147 (Figure S7), indicating the lipase was immobilized in the PAFs network as expected. The SEM and TEM images of lipase@PAF147 (Figure S8) showed after the immobilization of lipase the spherical morphology of the PAFs material remained without change. To further investigate the spatial distribution of lipases in PAF-147, green fluorescein labeled active ester (FITC-NHS) tagged lipase was applied and the confocal laser scanning microscopy (CLSM) was conducted. The 2D CLSM images demonstrated that lipases were exclusively located in PAF-147 under fluorescent irradiation (Figure 3b and Figure S9). The 3D CLSM image of lipase@PAF-147 is shown in Figure 3a, indicating the lipases were not only distributed on the surface of PAF-147 but also inside the particles. The TGA curve was shown in Figure S10, and the weight loss of lipase@PAF-147 was divided into two distinct steps. The weight loss was about 42% in the first step at 30–450 °C, which was attributed to the decomposition of lipase. The second step at 450–550 °C was assigned to the collapse of the material skeleton, and its weight loss was about 57%. The element analysis result was shown in Table S3. Compared to PAF-147, the N element was detected from the sample of lipase@PAF-147, with the ratio at 4.2% and the C element ratio was 75%. The detected N element from lipase@PAF-147 demonstrated that lipase was successfully loaded in PAF-147. After lipase loading, the N_2_ adsorption isotherm of lipase@PAF-147 exhibited a shape uptake at low relative pressure. Compared to PAF-147, the regulated typical I curve of lipase@PAF-147 indicated the retaining of the microporous structure, which was due to the rejection of large enzyme molecules by the micropores (Figure 4a). The specific surface area and pore volume of the loaded material were decreased from 2797 to 424 m^2^ g^–1^, and 1.92 cc g^–1^ to 0.36 cc g^–1^ respectively after lipase loading. The mesopores were not found after loading according to the NLDFT calculation. Only the micropores centered at 1.0 nm were retained (Figure 4b and Table S4). The UV–vis absorption spectra of the lipase’s supernatant filtrates after loading were shown in Figure S11, and the lipase loading capacity in PAF-147 was calculated to be 1456 mg g^–1^ through bicinchoninic acid (BCA) testing.^30^ These results indicated that hierarchical MTV-PAFs were efficient for enzyme loading due to the existence of the mesoporous structure, although the size of enzyme was slightly larger than the mesoporous structure, and this was consistent with what others have reported in the literature.^31,32^ Due to its high surface area and large pore volume, lipase@PAF-147 also showed the highest loading capacity compared with other reported enzyme-loading materials (Table S7).

**Figure 5:**
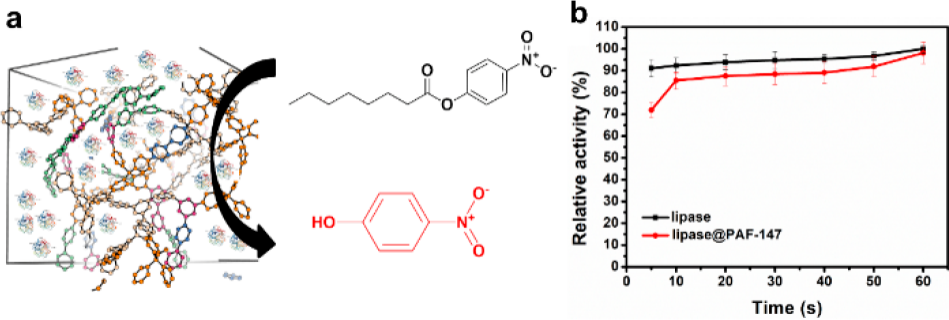
**a**, Schematic of the hydrolysis process of *p*-nitrophenyl caprylate to 4-nitrophenol. **b**, The relative activity of lipase and lipase@PAF-147 within 1 min at room temperature.

**Figure 6:**
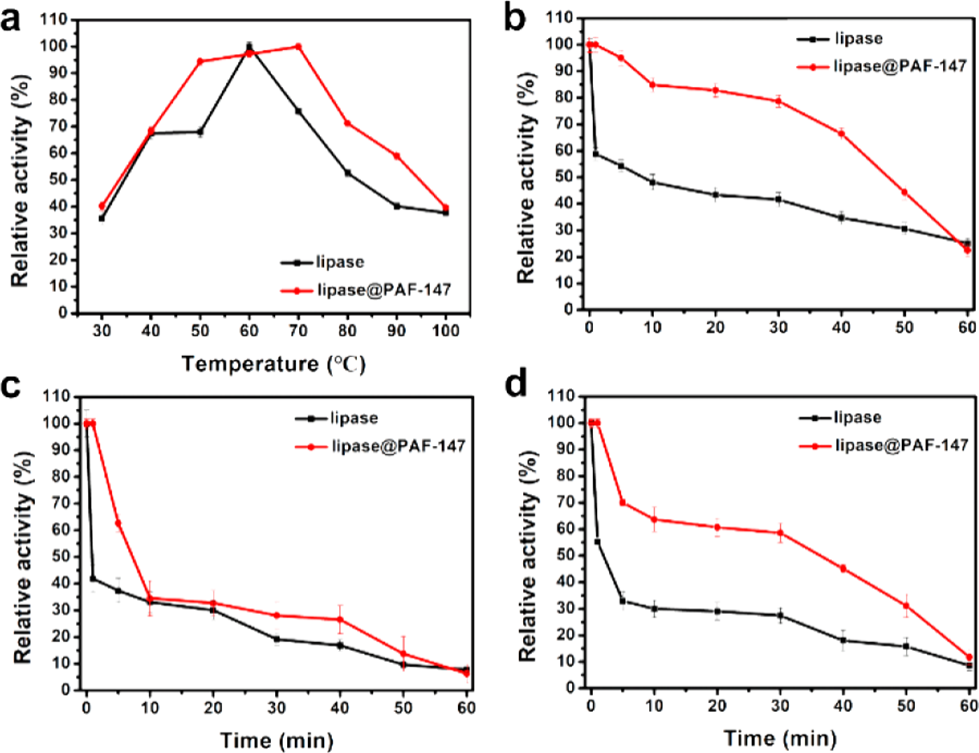
Relative catalytic activity of lipase and lipase@PAF-147 at **a**, 30 to 100 °C, neutral, **b**, 70 °C, neutral, **c**, 70 °C, acidic, pH = 4.0 and **d**, 70 °C, alkaline, pH = 10.0.

Enzymes are expensive and difficult to recycle. Their catalytic activity is easily affected by conditions such as temperature and pH value. The immobilization of enzymes in porous materials is able to solve these problems, and is beneficial to the future development of enzymes in industry. In principle, the lipase efficiently catalyzes of the conversion *p*-nitrophenyl caprylate (*p*-NPC) to 4-nitrophenol (Figure 5a), leading to a fast color change of the colorless solution to yellow solution within several seconds (Figure S13a). When lipase@PAF-147 was used to catalyze the hydrolysis of *p*-NPC to 4-nitrophenol, the color change of the *p*-NPC solution was also completed in seconds (Figure S13b), and the relative catalytic activity of lipase@PAF-147 was not reduced within 1 min after enzyme loading (Figure 5b). According to the calculation by the Michaelis–Menten model, the kinetic parameters (*K*_M_ and *V*_max_) of lipase@PAF-147 were 2.6357 mM and 0.09305 μM min^–1^, which was nearly close to lipase of 2.6537 mM and 0.09359 μM min^–1^. The results proved that lipase@PAF-147 has a good catalytic ability after loading enzyme (Figure S14 and Table S8). In general, enzyme immobilization increases the enzyme stability to the external conditions such as temperature and pH. The temperature profiles for lipase and lipase@PAF-147 are shown in Figure 6a. Neat lipase exhibited increased relative activity from 30 to 60 °C. At a temperature above 60 °C, the relative activity of lipase showed a significant decrease, indicating its poor temperature tolerance upon heating. In comparison, the relative activity of lipase@PAF-147 at the temperature range from 50 to 70 °C was above 95%, indicating the good temperature tolerance of lipase immobilized in PAFs. PAF-147 was able to protect lipase from heating, which was beneficial to expand the development of catalytic applications of lipase at a high temperature. As shown in Figure 6b–d, the pH tolerance of lipase and lipase@PAF-147 was also explored at 70 °C. Neutral, acidic, and alkaline environments were achieved by buffer solution with a pH of 7.0, 4.0, and 10.0, respectively. As shown in Figure 6b, at neutral condition, lipase@PAF-147 retained 50% relative activity after 47 min. In contrast, the relative activity of lipase rapidly decreased to 50% within 8 min. The results indicated that the half-life (*T*_1/2_) of lipase@PAF-147 had increased nearly six times longer than lipase, which revealed that lipase@PAF-147 was more stable at neutral environments. Moreover, lipase was sensitive to acid and base environments, and its catalytic activity was greatly reduced. For example, under acidic conditions (Figure 6c, pH = 4.0), the relative activity of lipase dropped very fast and its *T*_1/2_ was 0.77 min. At the same condition, the *T*_1/2_ of lipase@PAF-147 was 7.26 min, which was improved nearly 9 times after enzyme immobilization. A similar result was observed under an alkaline environment at pH of 10.0. The lipase@PAF-147 retained 50% of relative activity after 36 min under an alkaline environment (Figure 6d, pH = 10.0), while lipase only held 50% activity at 1.8 min. The *T*_1/2_ was improved nearly 20 times due to PAF protection. All these results are summarized in Table S9. These results proved that the immobilization of lipase could greatly improve its tolerance to the external condition like an acidic or alkaline environment, and this phenomenon was similar to the previously reported literature. The large pore in dual-pore COF could improve tolerant to detrimental byproducts.^33^ Moreover, the recyclability of lipase@PAF-147 was examined, and the results are shown in Figure S15. The lipase@PAF-147 showed almost steady relative activity through washing and regeneration. After four cycles, lipase@PAF-147 still had a relative activity above 90%, indicating the lipase@PAF-147 was well recycled. Therefore, a leaching experiment of lipase@PAF-147 was conducted, the results are shown in Figure S16, and the concentration of lipase released was lower than 0.086 mg/mL. These results indicated that their cost could be reduced by multiple usages.

## 3 Conclusions

In summary, a series of PAFs with a highly porous and hierarchical structure have been successfully synthesized through a multivariate strategy. Multiple building units with various shapes and sizes were combined and cross-linked together to give porous organic frameworks with a high surface area. Moreover, due to the existence of large building units, the PAFs also possessed uniformly distributed micropores of 1 nm and mesopores of 4–5 nm. The PAFs retained as good a stability as other reported PAFs. Encouraged by their hierarchical structures, we consider them to be suitable for enzyme immobilization. The lipase from *Aspergillus oryzae* was loaded in the PAF-147, and the given lipase@PAF-147 exhibited good stability and reusability for ester hydrolysis. The enzyme activity test suggested the lipase@PAF-147 elevated the stability of enzyme to external conditions, and specifically exhibited a 20 times higher half-life than the neat lipase at a pH of 10.0. Thus, this synthetic method provides an idea to construct PAFs with high porosity and hierarchical structures, and is also prospective to introduce structural and functional heterogeneity for designed PAFs synthesis in the future.

## 4 Experimental Section

### Preparation of MTV-PAFs

The synthetic route of PAFs is shown in Figure 1. A detailed version of the synthesis ratio is recorded in Table S1. For example, bis(1,5-cyclooctadiene) nickel (0) ([Ni(cod)_2_]) and 2,2′-bipyridyl were added to anhydrous DMF, and then 1,5-cyclooctadiene (cod) was added to a clear solution. The mixture solution was heated at 80 °C for 1 h. Then, another mixture solution of multiple building units was added to the above catalyst system by ratio, and the mixture solutions were stirred for 48 h. After that, when the mixture solution was cooled to the room temperature, concentrated hydrochloric acid was added to mixture. After filtration, the residue was washed with deionized water, and then Soxhlet was extracted with THF for 48 h to obtain a white powder, named PAF147, PAF-148, and PAF-149. All the reaction steps were carried out under a strict anaerobic environment.

## Supporting Information Available

Linker characterization, SEM, TEM, CLSM, TGA curves, and adsorption isotherms (PDF)
