# Supplementary material for: Multivariate Porous Aromatic Frameworks with High Porosity and Hierarchical Structures for Enzyme Immobilization
Source: ACS Cent Sci. 2023 Feb 16;9(3):488–93. doi: 10.1021/acscentsci.3c00078 (PMC10037458; doi:10.1021/acscentsci.3c00078)
Supplement: Supplementary file 1 — oc3c00078_si_001.pdf [file oc3c00078_si_001.pdf]

Supporting Information

Multivariate Porous Aromatic Frameworks with High Porosity and Hierarchical Structures for Enzyme Immobilization

Zhaofu Zhang,[†] *Yajing Zheng,*[†] *Zilong Dou,*[†] *Mengnan Gu,*[†] *Mengxiao Sun,*[†] *Jian Song,*[†] *Nan Gao,*[†] *Fengchao Cui,*[†] *Yuyang Tian,**[,†] *and Guangshan Zhu**[,†]

[†] *Key Laboratory of Polyoxometalate and Reticular Material Chemistry of Ministry of Education, Faculty of Chemistry, Northeast Normal University, Changchun, 130024, China*
E-mail: tianyy100@nenu.edu.cn and zhugs@nenu.edu.cn

Keywords: Multivariate Porous Aromatic Framework; Highly Surface Area; Hierarchical Structures; Enzyme Immobilization; Catalysis

1 Materials and methods S2
2 Synthetic procedure S2
2.1 Synthesis of PAF-147, PAF-148 and PAF-149 .... S2
2.2 Synthesis of Compound 1, Compound 2 and Compound 3 .... S3
2.3 Synthesis of lipase@PAF-147 .... S3
2.4 Calculation of adsorption capacity for lipase@PAF-147 .... S3
2.5 Ester hydrolysis catalysis experiment of lipase@PAF-147 .... S4
3 Characterizations S5
References S15

1 Materials and methods

Materials

1,4-Dibromobenzene, 4,4-Dibromobiphenyl, 1,3,5-Tribromobenzene, 1,5-Cyclooctadiene, 2,2'-Bipyridine, N, N-Dimethylformamide and Bis(1,5-cyclooctadiene)nickel(0) were purchased from Innochem. 1,3,5-Tribromophenylbenzen and Tetra(4-bromophenyl)methane were purchased from Jilin Chinese Academy of Sciences-Yanshen Technology Co., Ltd. Lipase from Aspergillus oryzae and p-nitrophenyl caprylate (p-NPC) were purchased from Shanghai Kanglang Biological Technology Co., Ltd. The Micro-BCA Protein Assay Kit was obtained from Beyotime Biotech Inc. The FITC-NHS was purchased from Xian ruixi Biological Technology Co., Ltd. All above mentioned solvents and chemicals were obtained from commercial sources and used without further purification unless otherwise noted.

General instrumentations and methods

The FT-IR spectra were collected on a FT-IR spectrometer (Nicolet IS50), over the wavenumber range was 400-4000 cm^{-1}. The micromorphology and elemental mapping of samples were implemented on the field emission scanning electron microscope (FE-SEM, SU-8010, Hitachi) and transmission electron microscope (TEM, JEOL 2100PLUS). The CLSM images of lipase@PAF-147 was conducted by laser confocal microscope (Olympus FV1200). The elemental analyses of materials (for C, H and N) were measured with a Vario MACRO cube CHNS. Thermogravimetric analysis was performed on thermogravimetric analyzer METTLER-TOLEDO TGA/DSC 3+, samples were heated from 30 to 800 ℃ at the 10 ℃/min under air atmosphere. The surface area and pore size distribution of samples were obtained using an Autosorb iQ2 adsorptometer, Quantachrome Instrument at 77 K. The relative activity of lipase and lipase@PAFs were tested by UV-visible spectrophotometer.

2 Synthetic procedure

2.1 Synthesis of PAF-147, PAF-148 and PAF-149

The synthetic route of PAFs is shown in Figure 1. A detailed version of the synthesis ratio is recorded in the Table S1. For example, the bis(1,5-cyclooctadiene)nickel(0) ([$Ni(cod)_2$]) and 2,2'-bipyridyl was added to anhydrous DMF, then the 1,5-cyclooctadiene (cod) was added to a clear solution. The mixture solution was heated at 80 ℃ for 1 h. Then, another mixture solution about 1,4-Dibromobenzene, 4,4-Dibromobiphenyl, 1,3,5-Tribromobenzene, 1,3,5-Tribromophenylbenzen and Tetra(4-bromophenyl)methane was added to above catalyst system by ratio, and the mixture solutions were stirred for 48 h. After that, when the mixture solution was cooled to the room temperature, the

concentrated hydrochloric acid was added to mixture. After filtration, the residue was washed with deionized water, then Soxhlet extracted with THF for 48 h to obtain white powder, named PAF147, PAF-148 and PAF-149. All the reaction steps were carried out under a strict anaerobic environment.

2.2 Synthesis of Compound 1, Compound 2 and Compound 3

The Compound 1 and Compound 2 were obtained by Ullman coupling. Compound1 was synthesized with equal molar amounts of 1,4-Dibromobenzene and Tetra(4-bromophenyl)methane (i.e., 50% of each), and Compound 2 was synthesized by equal molar amounts of 1,3,5-Tribromophenylbenzen and Tetra(4-bromophenyl)methane (i.e., 50% of each). The subsequent treatment was the same as that of PAF-147, but their yields were only 52.6% and 74.5%, which was lower than the normal yield of PAF-147, PAF-148 and PAF-149, presumably because linear or triangular building units were not dispersed enough during the reaction, and self-polymerization occurred to reduce the product. The Compound 3 was also constructed with 1,4-dibromotetrafluorobenzene, 2,4,6-tribromoaniline, 4,4-dibromo-2-nitrobiphenyl, 1,3,5-tris(4-bromophenyl) phosphorus and tetrabromotetraphenylsilane, and the Compound 3 was used for mapping test.

2.3 Synthesis of lipase@PAF-147

First, 50 mg PAF-147 was selected to disperse in 10 ml lipase solution (46.475 mg/ml) and stirring for 12 h under low temperature of 2-4 ℃. Then materials were collected through centrifugal and washed their surface with deionized water until no catalytic activity could be detected in the washing solution. And the materials were vacuum dried at room temperature to obtain lipase@PAF-147. The protein concentration of lipase was determined through bicinchoninic acid (BCA) method using the Micro-BCA Protein Assay Kit.

2.4 Calculation of adsorption capacity for lipase@PAF-147

The amount of lipase loaded was calculated as the following formula:

Lipase loading = (A-B)*C/D

where A and B was the protein concentration of lipase in the initial solution (46.475 mg/ml) and residual solution (39.194 mg/ml), respectively, C was the volume of lipase system (10 ml) and D was quality of PAF-147 for loading (50 mg). In addition, the protein concentration of lipase in the initial solution and residual solution were determined through bicinchoninic acid (BCA) method using the Micro-BCA Protein Assay Kit.

2.5 Ester hydrolysis catalysis experiment of lipase@PAF-147

In order to investigate the catalytic activity, stability and reusability of lipase@PAF-147, the *p*-nitrophenyl caprylate (*p*-NPC) was selected as model to research these problems. The relative activity of lipase@PAF-147 was calculated to measure the absorbance of the generated *p*-nitrophenol at 400 nm with ultraviolet spectrophotometer. First, the 10 mM *p*-NPC solution was prepared with acetonitrile. Then, 20 μL lipase solution (46.475 mg/ml) was added to 960 μL phosphate buffer (pH=7.0) and treated at predetermined temperature (30 ℃, 40 ℃, 50 ℃, 60 ℃, 70 ℃, 80 ℃, 90 ℃ and 100 ℃) for 1 min, and 20 μL *p*-NPC solution was added to initiate reaction. Similarly, 0.5 mg lipase@PAF-147 and 980 μL phosphate buffer (pH=7.0) were treated at same temperature for 1 min, then 20 μL *p*-NPC solution was added. After that, the supernatant filtrate was measured with ultraviolet spectrophotometer. And the tolerance of high temperature and pH stability of lipase and lipase@PAF-147 were systematically investigated with similar way. First, 20 μL lipase (46.475 mg/ml), 960 μL PBS (pH=7.0) or 0.5 mg lipase@PAF-147, 980 μL PBS (pH=7.0) were investigated through the pre-incubation for different time (1 min, 5 min, 10 min, 20 min, 30 min, 40 min, 50 min, 60 min) at 70 ℃, then 20 μL *p*-NPC solution was added, the testing process was similar to previous step. And we adjusted the system to be acidic (pH=4.0) and alkaline (pH=10.0) for the same measure process, it is worth noting that the pH values should adjust at 70 ℃. The cycle experiments were also conducted through same method. The leaching experiment of lipase@PAF-147 was also carried out. 10 mg lipase@PAF-147 was left in 20 ml PBS buffer solution for 6 months, then the supernatant was taken for BCA protein test. The K_M and V_{max} were determined by hydrolyzing under different concentrations ([S]=1, 1.25, 2, 2.5, 4.5 and 5 mM). The result was measured spectrophotometrically, and the Beer-Lambert's law was applied to determine the content of product to get v for [S]. Then $1/v$ and $1/[S]$ were fitted in a Lineweaver-Burk plot to give K_M and V_{max}. In addition, the background hydrolysis absorbance of *p*-NPC has been deducted using the blank control group (without lipase solution and lipase@PAF-147).

3 Characterizations

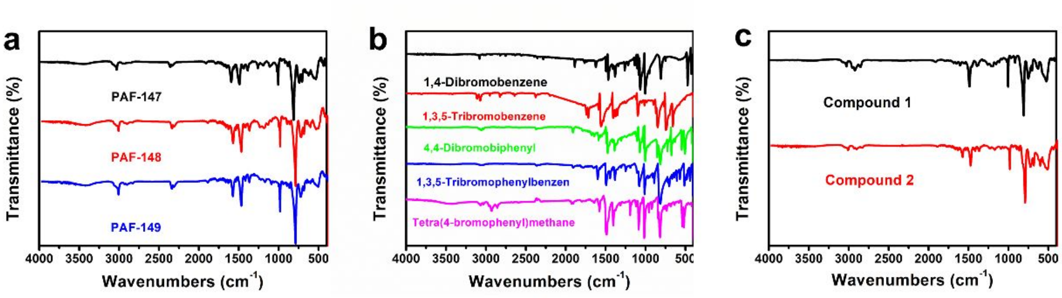

Figure S1. The FT-IR spectra of (a) PAF-147, PAF-148 and PAF-149, (b) five building units, (c) Compound 1 and Compound 2.

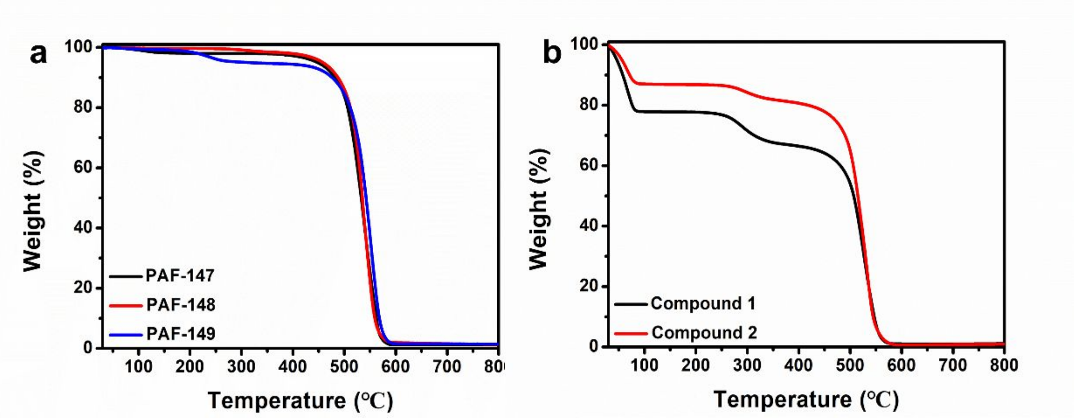

Figure S2. The TG curves of (a) PAF-147, PAF-148 and PAF-149, (b) Compound 1 and Compound 2.

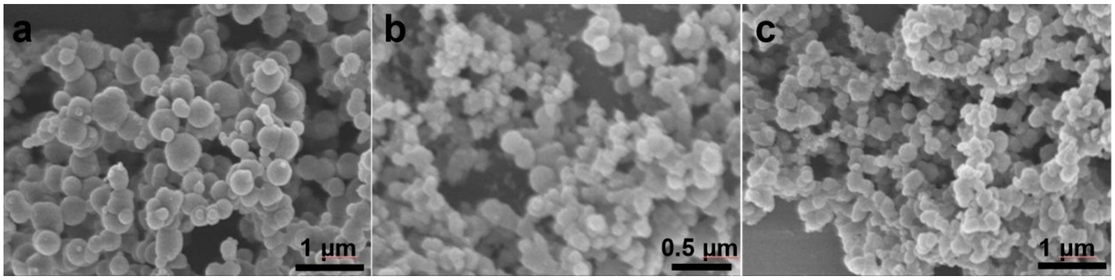

Figure S3. The SEM images of (a) PAF-147, (b) PAF-148 and (c) PAF-149.

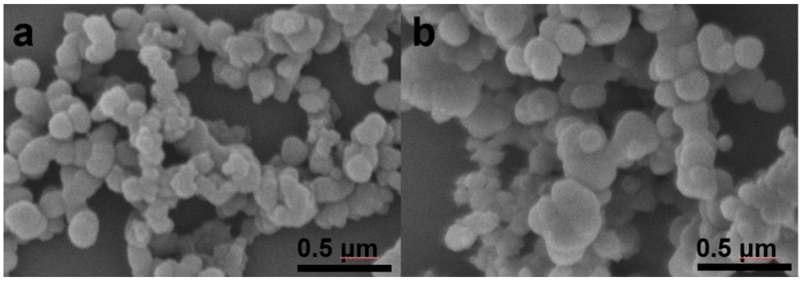

Figure S4. The SEM images of (a) Compound 1 and (b) Compound 2.

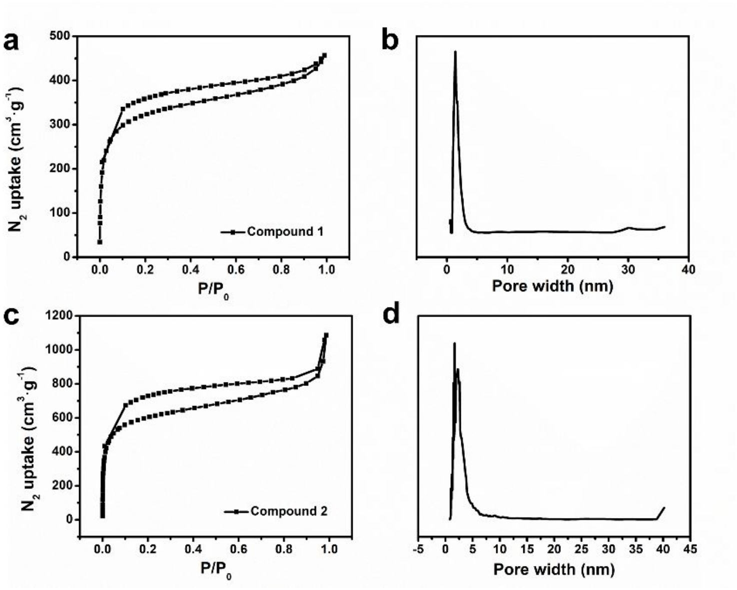

Figure S5. The N_2-sorption isotherm of Compound 1 and Compound 2.

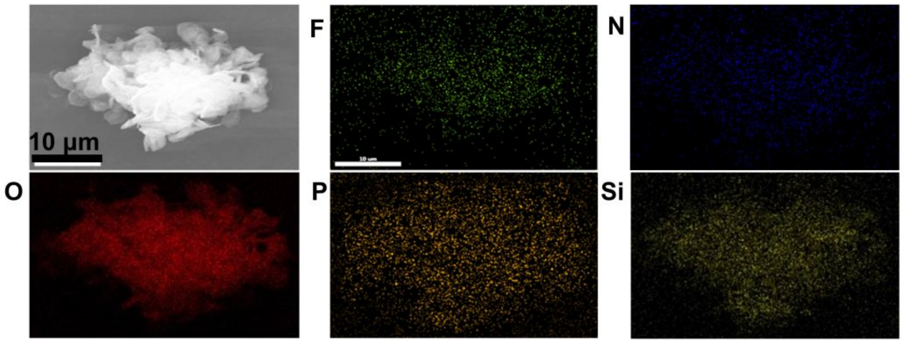

Figure S6. The SEM mapping images of Compound 3.

The Compound 3 was constructed by five building units with different elements, and the mapping tests was toke, the results were shown in Figure S6, the five elements (F, N, O, P, Si) were randomly and evenly distributed in material structure. This proved that multiple building units were evenly distributed during PAF synthesis to better connect with each other.

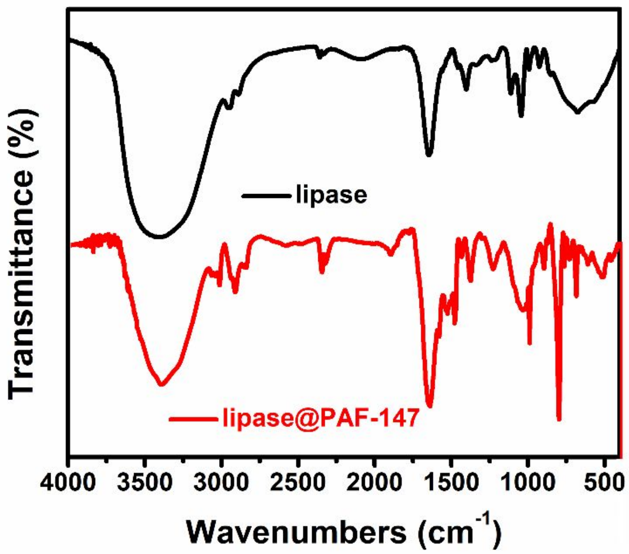

Figure S7. The FT-IR spectrum of lipase and lipase@PAF-147.

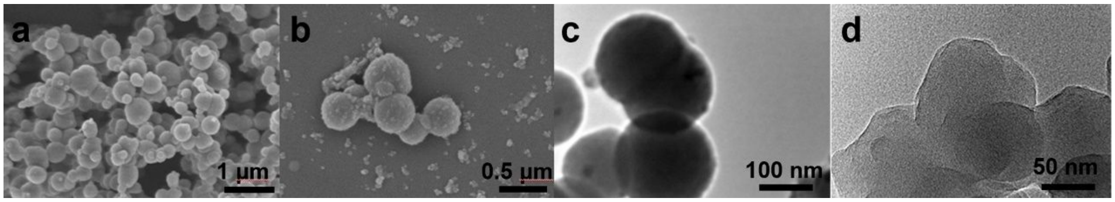

Figure S8. The SEM images of (a) PAF-147, (c) lipase@PAF-147 and TEM images of (b) PAF-147, (d) lipase@PAF-147.

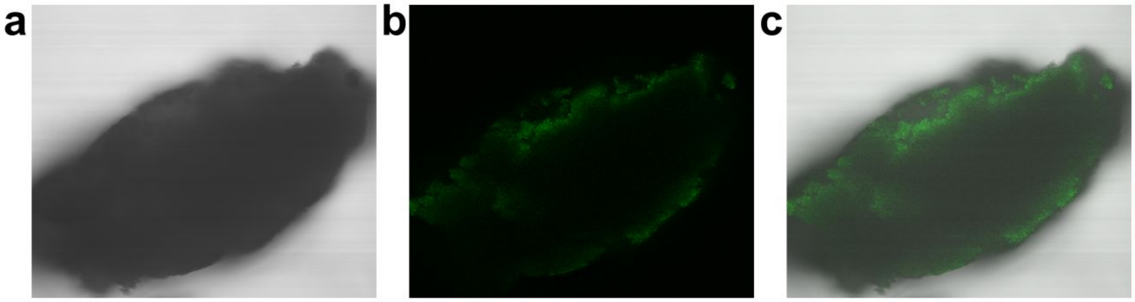

Figure S9. The 2D CLSM images of lipase@PAF-147 scale bar: 30 μm (a: white light, b: fluorescence, c: white light+fluorescence).

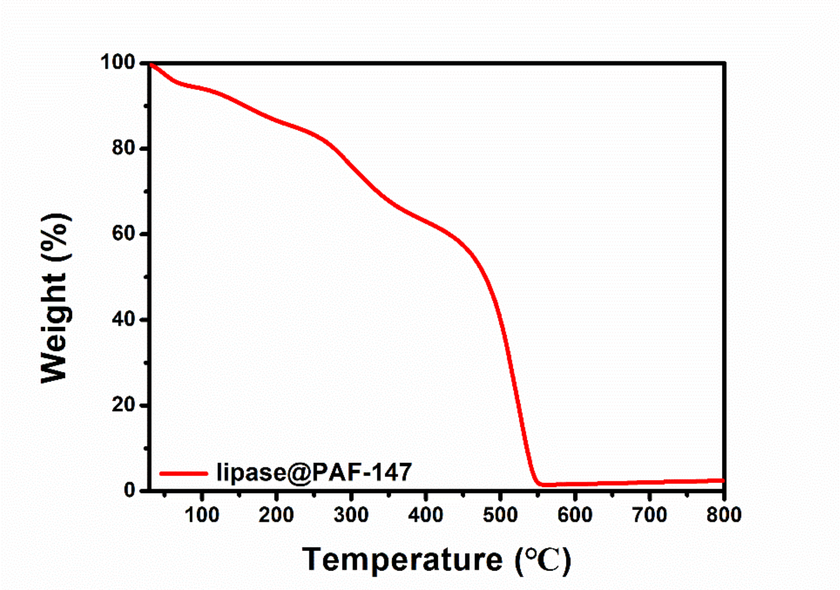

Figure S10. The TG curves of lipase@PAF-147.

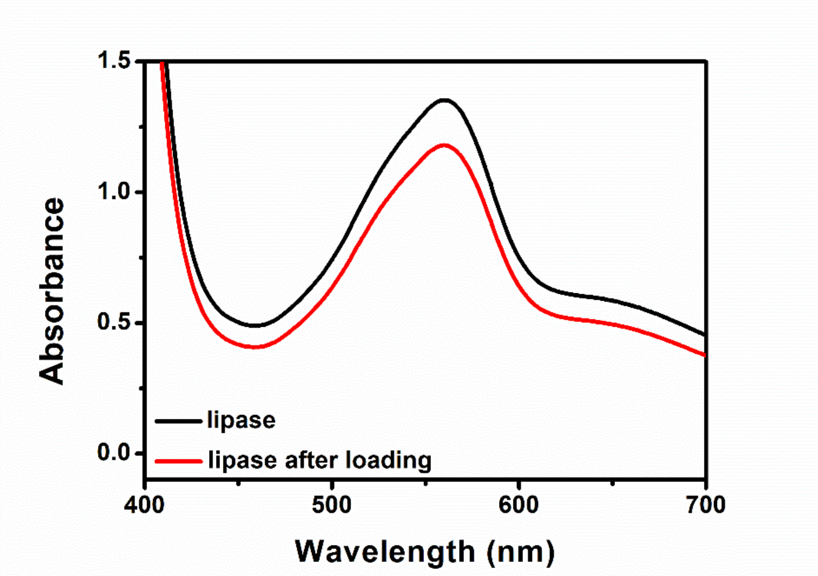

Figure S11. The UV absorption spectrum of lipase and lipase after loading by PAF-147.

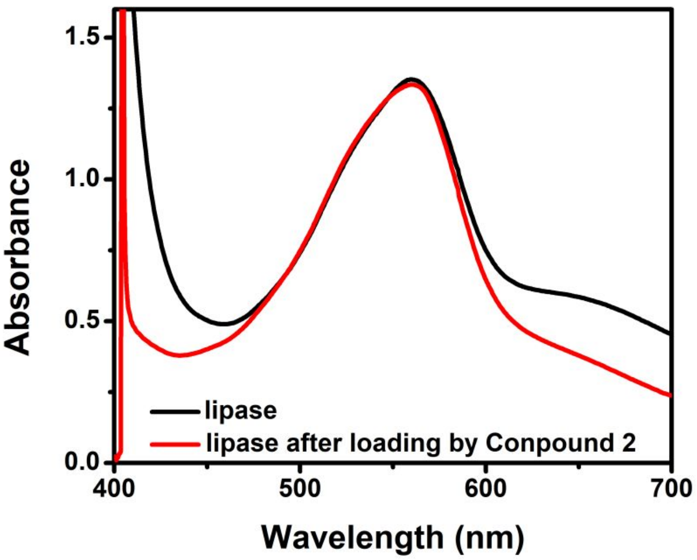

Figure S12. The UV absorption spectrum of lipase and lipase after loading by Compound 2.

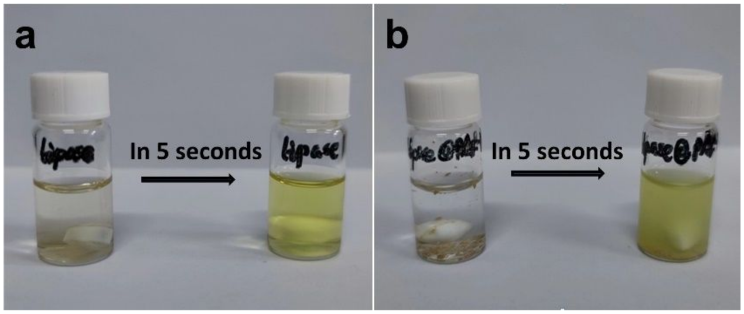

Figure S13. The optical images of (a) lipase and (b) lipase@PAF-147 catalytic process.

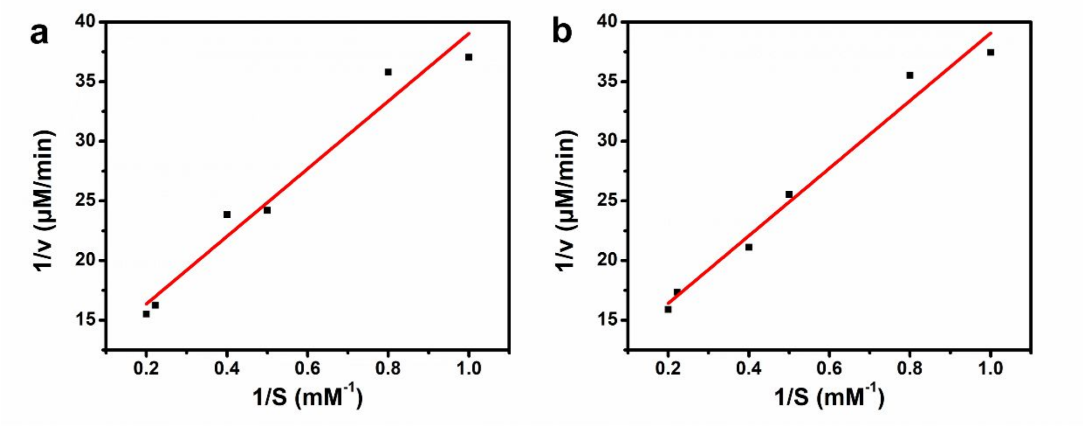

Figure S14. A Lineweaver-Burk plot of hydrolysis experiments for *p*-NPC catalyzed by lipase and lipase@PAF-147 based on the Michaelis-Menten model.

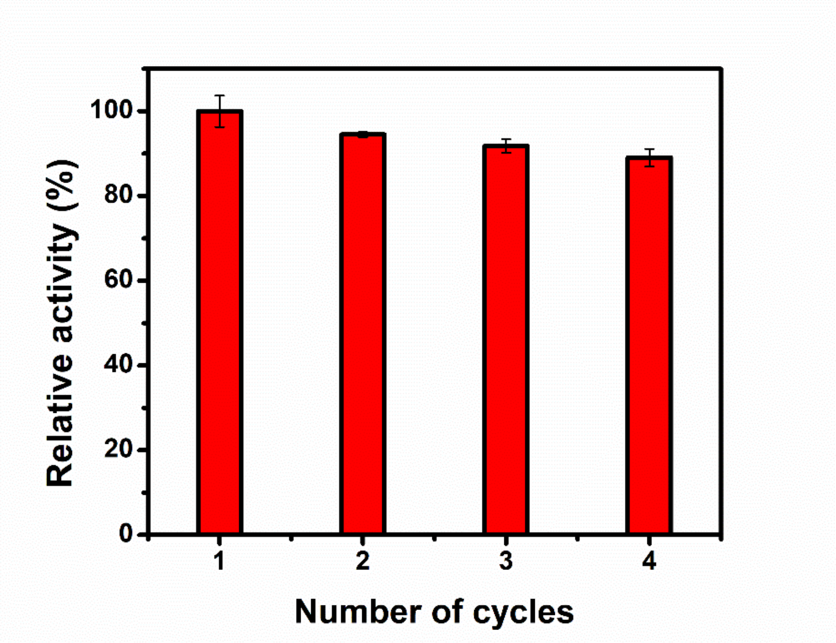

Figure S15. The cycle performance of and lipase@PAF-147.

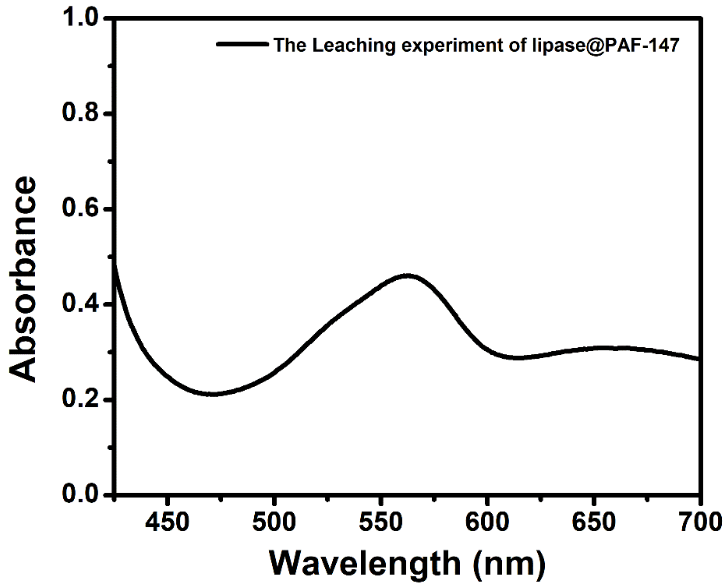

Figure S16. The leaching experiment of lipase@PAF-147.

Table S1.

	1,4-Dibromobenzene	4,4-Dibromobiphenyl	1,3,5-Tribromobenzene	1,3,5-Tribromophenylbenzen	Tetra(4-bromophenyl)methane	Yield
PAF-147	20%	20%	20%	20%	20%	85.9%
PAF-148	30%	30%	10%	10%	20%	85.3%
PAF-149	10%	10%	30%	30%	20%	85.8%
Compound 1	50%	0		0	50%	52.6%
Compound 2	0	0	0	50%	50%	74.5%

Table S2.

	C (%)	H (%)	N (%)
PAF-147	93.144	2.449	-
PAF-148	90.125	3.087	-
PAF-149	91.127	3.847	-

Table S3.

	C (%)	H (%)	N (%)
PAF-147	93.144	2.449	-
lipase@PAF-147	75.633	5.635	4.253

Table S4.

	BET ($m^2\ g^{-1}$)	Pore width (nm)	Pore volume (cc g^{-1})
PAF-147	2797	1.0, 4.2	1.9
PAF-148	2877	1.0, 4.5	2.0
PAF-149	2856	1.0, 4.1	2.2
lipase@PAF-147	424	1.0	0.36

Table S5.

	BET ($m^2\ g^{-1}$)	Pore width (nm)	Pore volume (cc g^{-1})
Compound 1	1185	1.4	0.7
Compound 2	2218	1.6, 2.2	1.6

Table S6.

Materials	Pore size (nm)	BET ($m^2\ g^{-1}$)	Ref	Materials	Pore size (nm)	BET ($m^2\ g^{-1}$)	Ref
PAF-11	0.5/5	704	1	Py-MV-DBA-COF	4.1	1134	17
PAF-70-NH2	1.5/3.8	599	2	FLT-COF-1	3.7	1180	18
PPN-4/C600	4.7	1323	3	COF-DhaTab	3.7	1480	19
PPN-4/C600-Si	5.0	636	3	TAPB-TFPB	4.0	229	20
JUC-Z15	1.41/4.0	1570	4	4PE-2P COF	3.8	2070	21
COF-10	3.41	2080	5	COF-BPDA	1.27/3.18	477.76	22
TP-COF	3.26	868	6	TPB-DMTP-	3.3	1927	23

				COF			
T-COF 3	3.15	544	7	TAPT-DHTA-COF	3.1	2170	24
HHTP-DPB COF	4.7	930	8	TFPT-COF	3.4	1603	25
DBA-COF 1	3.4	1952	9	PI-COF-2	3.3	1297	26
AEM-COF-1	3.2	1445	10	PI-COF-3	5.1	2346	26
TP-Por-COF	4.6	890	11	3'PD	3.19/4.52	258	27
ZnPc-PPE COF	3.4	440	12	CuPc-FPBA-TMBDA	3.11	1141	28
DZnPc-ANDI-COF	3.6	1410	13	HHTP-FFPBA-TATTA	3.17	1748	28
DCuPc-APDI-COF	4.0	414	14	PAF-147	1.0/4.2	2797.3	This work
DTP-ANDI-COF	5.3	1910	15	PAF-148	1.0/4.5	2877.5	This work
Star-COF-2	4.4	1767	16	PAF-149	1.0/4.1	2856.8	This work
Star-COF-3	4.7	2129	16	Compound 1	1.4	1185.1	This work
Py-DBA-COF 1	3.6	1392	17	Compound 2	1.6/2.2	2218.2	This work

Table S7.

Materials	BET (m^2 g^{-1})	Pore size (nm)	Capacity (μmol g^{-1})	Capacity (mg g^{-1})	References
MP-11@mesoMOF	1935	0.9/3.0/4.1	19.1	37.70	29
MP-11@MCM-41	1000	2-5	3.4	6.711	29

H-IgG@ZIF-90	1270	0.35	-	530	30
H-IgG@ZIF-8	1836	0.34-1.10	-	370	30
G-IgG@ZIF-90	1270	0.35	-	590	30
G-IgG@ZIF-8	1836	0.34-1.10	-	420	30
Mb@Tb-mesoMOF	1935	0.9/3.0/4.1	9.1	151.97	31
Cytc@TbmesoMOF	1935	0.3/3.0/4.1	10.2	657.9	32
BSA@ZPF-1	-	-	-	236.5	33
Cytc@NKMOF101	-	-	-	229.9-389	34
lipase@NKMOF101	-	-	-	97-168	34
BSA@ZIF-90	1270	0.35	-	220	35
GOx@PCN-888	3700	6.2	-	1000	36
HRP@PCN-333	4000	5.5	-	1000	36
Cytc@PCN-333	4000	5.5	-	950	37
MP-11@PCN-333	4000	5.5	-	870	37
OPAA@PCN-128y	-	4.4	-	120	38
Compound 2	2218	1.6/2.2		200.6	This work
lipase@PAF-147	2797	1.0/4.2	-	1456.06	This work

Table S8.

	K_M	V_{max}

lipase	2.6537 mM	0.09359 μM min^{-1}
lipase@PAF-147	2.6357 mM	0.09305 μM min^{-1}

Table S9.

	lipase	lipase@PAF-147	multiple
$T_{1/2}$	8.44 min	47.49 min	5.62
$T_{1/2}$	0.77 min	7.26 min	9.42
$T_{1/2}$	1.86 min	36.29 min	19.51

References

[1] Y. Yuan, F. Sun, H. Ren, X. Jing, W. Wang, H. Ma, H. Zhao, G. Zhu, Targeted synthesis of a porous aromatic framework with a high adsorption capacity for organic molecules. *J. Mater. Chem.* **2011**, *21*, 13498-13502.

[2] J. Sun, L. Jing, Y. Tian, F. Sun, P. Chen, G. Zhu, Task-specific design of a hierarchical porous aromatic framework as an ultrastable platform for large-sized catalytic active site binding. *Chem. Commun.* **2018**, *54,* 1603-1606.

[3] B. Li, Y. Zhang, D. Ma, L. Zhu, D. Zhang, M. Chrzanowski, Z. Shi, S. Ma, Creating extra pores in microporous carbon via a template strategy for a remarkable enhancement of ambient-pressure CO 2 uptake. *Chem. Commun*. **2015**, *51*, 8683-8686.

[4] C. Lu, T. Ben, S. Qiu. Macromol, Synthesis and gas storage application of hierarchically porous materials. *Chem. Phys*. **2016**, *217*, 1995-2003.

[5] A. Cote, H. El-Kaderi, H. Furukawa, J. Hunt, O. Yaghi, Reticular synthesis of microporous and mesoporous 2D covalent organic frameworks. *J. Am. Chem. Soc*. **2007**, *129*, 12914-12915.

[6] S. Wan, J. Guo, J. Kim, H. Ihee, D. Jiang, A belt - shaped, blue luminescent, and semiconducting covalent organic framework. *Angew. Chem. Int. Ed.* **2008**, *47*, 8826-8830.

[7] G. Bertrand, V. Michaelis, T. Ong, M. Dinca, Thiophene-based covalent organic frameworks. *PNAS* **2013**, *110*, 4923-4928.

[8] E. Spitler, B. Koo, J. Novotney, J. Colson, F. Uribe-Romo, G. Gutierrez, P. Clancy, W. Dichtel, A 2D covalent organic framework with 4.7-nm pores and insight into its interlayer stacking. *J. Am. Chem. Soc*. **2011**, *133*, 19416-19421.

[9] L. Baldwin, J. Crowe, M. Shannon, C. Jaroniec, P. Mcgrier, 2D covalent organic frameworks with alternating triangular and hexagonal pores. *Chem. Mater.* **2015**, *27*, 6169-6172.

[10] H. Yang, Y. Du, S. Wan, G. Trahan, Y. Jin, W. Zhang, Mesoporous 2D covalent organic frameworks based on shape-persistent arylene-ethynylene macrocycles. *Chem. Sci*. **2015**, *6*, 4049-4053.

[11] M.Calik, F. Auras, L. Salonen, K. Bader, I. Grill, M. Handloser, D. Medina, M. Dogru, F. Lobermann, D. Trauner, A. Hartschuh, T. Bein, Extraction of photogenerated electrons and holes from a covalent organic framework integrated heterojunction. *J. Am. Chem. Soc*. **2014**, *136*, 17802-17807.

[12] E. Spitler, J. Colson, F. Uribe-Romo, A. Woll, M. Giovino, A. Saldivar, W. Dichtei, Lattice expansion of highly oriented 2D phthalocyanine covalent organic framework films. *Angew. Chem. Int. Ed.* **2012**, *124*, 2677-2681.

[13] S. Jin, X. Ding, X. Feng, M. Supur, K. Furukawa, S. Takahashi, M. Addicoat, M. EI-Khouly, T. Nakamura, S. Irle, S. Fukuzumi, A. Nagai, D. Jiang, Charge Dynamics in A Donor–Acceptor Covalent Organic Framework with Periodically Ordered Bicontinuous Heterojunctions. *Angew. Chem. Int. Ed.* **2013**, *125*, 2071-2075.

[14] S. Jin, M. Supur, M. Addicoat, K. Furukawa, L. Chen, T. Nakamura, S. Fukuzumi, S. Irle, D. Jiang, Creation of Superheterojunction Polymers via Direct Polycondensation: Segregated and Bicontinuous Donor–Acceptor π-Columnar Arrays in Covalent Organic Frameworks for Long-Lived Charge Separation. *J. Am. Chem. Soc.* **2015**, *137*, 7817-7827.

[15] S. Jin, K. Furukawa, M. Addicoat, L. Chen, S. Takahashi, S. Irle, D. Jiang, Large pore donor–acceptor covalent organic frameworks. *Chem. Sci.* **2013**, *4*, 4505-4511.

[16] X. Feng, Y. Dong, D. Jiang, Star-shaped two-dimensional covalent organic frameworks. *CrystEngComm* **2013**, *15*, 1508-1511.

[17] J. Crowe, L. Baldwin, P. McGrier, Luminescent covalent organic frameworks containing a homogeneous and heterogeneous distribution of dehydrobenzoannulene vertex units. *J. Am. Chem. Soc.* **2016**, *138*, 10120-10123.

[18] C. Thompson, G. Occhialini, G. McCandless, S. Alahakoon, V. Cameron, S. Nielsen, R. Smaldone, Computational and experimental studies on the effects of monomer planarity on covalent organic framework formation. *J. Am. Chem. Soc.* **2017**, *139*, 10506-10513.

[19] S. Kandambeth, V. Venkatesh, D. Shinde, S. Kumari, A. Halder, S. Verma, R. Banerjee, Self-templated chemically stable hollow spherical covalent organic framework. *Nat. Commun*. **2015**, *6*, 1-10.

[20] J. Brédas, J. Norton, J. Cornil, V. Coropceanu, Molecular Understanding of Organic Solar Cells: The Challenges. *Acc. Chem. Res.* **2009**, *42*, 1691-1699.

[21] L. Ascherl, T. Sick, J. Margraf, S. Lapidus, M. Calik, C. Hettstedt, K. Karaghiosoff, M. Doblinger, T. Clark, K. Chapman, F. Auras, T. Bein, Molecular docking sites designed for the generation of highly crystalline covalent organic frameworks. *Nat. Chem*. **2016**, *8*, 310-316.

[22] Z. Pang, S. Xu, T. Zhou, R. Liang, T. Zhan, X. Zhan, Construction of covalent organic frameworks bearing three different kinds of pores through the heterostructural mixed linker strategy. *J. Am. Chem. Soc*. **2016**, *138*, 4710-4713.

[23] H. Xu, J. Gao, D. Jiang, Stable, crystalline, porous, covalent organic frameworks as a platform for chiral organocatalysts. *Nat. Chem.* **2015**, *7*, 905-912.

[24] Q. Xu, Y. Tang, X. Zhang, Y. Oshima, Q. Chen, D. Jiang, Template conversion of covalent organic frameworks into 2D conducting nanocarbons for catalyzing oxygen reduction reaction. *Adv. Mater*. **2018**, *30*, 1706330.

[25] L. Stegbauer, K. Schwinghammer, B. Lotsch, A hydrazone-based covalent organic framework for photocatalytic hydrogen production. *Chem. Sci.* **2014**, *5*, 2789-2793.

[26] Q. Fang, Z. Zhuang, S. Gu, R. Kaspar, J. Zheng, J. Wang, S. Qiu, Y. Yan, Designed synthesis of large-pore crystalline polyimide covalent organic frameworks. *Nat. Commun.* **2014**, *5*, 1-8.

[27] M. Rao, Y. Fang, S. Feyter, D. Perepichka, Conjugated covalent organic frameworks via michael addition–elimination. *J. Am. Chem. Soc*. **2017**, *139*, 2421-2427.

[28] X. Chen, M. Addicoat, E. Jin, H. Xu, T. Hayashi, F. Xu, N. Huang, S. Irle, D, Jiang, Designed synthesis of double-stage two-dimensional covalent organic frameworks. *Sci. Rep*. **2015**, *5*, 1-19.

[29] V. Lykourinou, Y. Chen, X. Wang, L. Meng, T. Hoang, L. Ming, R. Musselman, S. Ma, Immobilization of MP-11 into a mesoporous metal–organic framework, MP-11@ mesoMOF: a new platform for enzymatic catalysis. *J. Am. Chem. Soc.* **2011**, *133*, 10382-10385.

[30] Y. Feng, H. Wang, S. Zhang, Y. Zhao, J. Gao, Y. Zheng, P. Zhao, Z. Zhang, M. Zaworotko, P. Cheng, S. Ma, Y. Chen, Antibodies@MOFs: An In Vitro Protective Coating for Preparation and Storage of Biopharmaceuticals. *Adv. Mater.* **2019**, *31*, 1805148.

[31] Y. Chen, V. Lykourinou, T. Hoang, L. Ming, S. Ma, Size-selective biocatalysis of myoglobin immobilized into a mesoporous metal–organic framework with hierarchical pore sizes. *Inorg. Chem.* **2012**, *51*, 9156-9158.

[32] Y. Chen, V. Lykourinou, C. Vetromile, T. Hoang, L. Ming, R. Larsen, S. Ma, How Can Proteins Enter the Interior of a MOF? Investigation of Cytochrome c Translocation into a MOF Consisting of Mesoporous Cages with Microporous Windows. *J. Am. Chem. Soc*. **2012**, *134*, 13188-13191.

[33] H. Wang, L. Han, D. Zheng, M. Yang, Y. Andaloussi, P. Cheng, Z. Zhang, S. Ma, M. Zaworotko, Y. Feng, Y. Chen, Protein ‐ structure ‐ directed metal ‐ organic zeolite ‐ like networks as biomacromolecule carriers. *Angew. Chem. Int. Ed.* **2020**, *59*, 6263-6267.

[34] H. An, J. Song, T. Wang, N. Xiao, Z. Zhang, P. Cheng, S. Ma, H. Huang, Y. Chen, Metal–organic framework disintegrants: enzyme preparation platforms with boosted activity. *Angew. Chem. Int. Ed.* **2020**, *132*, 16907-16912.

[35] M. Li, S. Qiao, Y. Zheng, Y. Andaloussi, X. Li, Z, Zhang, A. Li, P. Cheng, S. Ma, Y. Chen, Fabricating covalent organic framework capsules with commodious microenvironment for enzymes. *J. Am. Chem. Soc.* **2020**, *142*, 6675-6681.

[36] X. Lian, Y. Chen, T. Liu, H. Zhou, Coupling two enzymes into a tandem nanoreactor utilizing a hierarchically structured MOF. *Chem. Sci.* **2016**, *7*, 6969-6973.

[37] D. Feng, T. Liu, J. Su, M. Bosch, Z. Wei, W. Wan, D. Yuan, Y. Chen, X. Wang, K. Wang, X. Lian, Z. Gu, J. Park, X. Zou, H. Zhou, Stable metal-organic frameworks containing single-molecule traps for enzyme encapsulation. *Nat. Commun*. **2015**, *6*, 1-8.

[38] P. Li, S. Moon, M. Guelta, S. Harvey, J. Hupp, O. Farha, Encapsulation of a nerve agent detoxifying enzyme by a mesoporous zirconium metal–organic framework engenders thermal and long-term stability. *J. Am. Chem. Soc.* **2016**, *138*, 8052-8055.
